# Microbial proteases and endothelial barrier disruption in sepsis: A neglected nexus

**DOI:** 10.1080/21505594.2026.2687236

**Published:** 2026-06-14

**Authors:** Shamitha S. Rao, Suchitra Shenoy M, Ravi Kumar Gutti, Vijendra Prabhu, Manjunath Joshi, Usha Yogendra Nayak, A. S. Bharath Prasad

**Affiliations:** a Department of Public Health Genomics, Manipal School of Life Sciences, Manipal Academy of Higher Education, Manipal, India; b Department of Microbiology, Kasturba Medical College, Manipal Academy of Higher Education, Mangalore, India; c Department of Biochemistry, School of Life Sciences, University of Hyderabad, Hyderabad, India; d Department of Biotechnology, Manipal Institute of Technology, Manipal Academy of Higher Education, Manipal, India; e Department of Ageing Research, Manipal School of Life Sciences, Manipal Academy of Higher Education, Manipal, India; f Department of Pharmaceutics, Manipal College of Pharmaceutical Sciences, Manipal Academy of Higher Education, Manipal, India

**Keywords:** Sepsis, endothelium, bacterial proteases, coagulopathy, barrier disruption

## Abstract

Sepsis is a life-threatening condition characterized by dysregulated host responses to infection and remains a leading cause of mortality globally. While host inflammatory pathways have been extensively studied, the contribution of bacterial proteases to sepsis pathogenesis remains underappreciated. Emerging evidence indicates that bacterial proteases act as potent virulence factors that directly target the vascular endothelium by cleaving junctional proteins, degrading the glycocalyx, inactivating anticoagulant molecules and degrading key coagulation factors such as fibrinogen, factor V, factor VIII and thrombin. This combined structural and functional damage leads to endothelial barrier failure, vascular leakage and progression toward disseminated intravascular coagulation (DIC). Additionally, bacterial proteases increase inflammatory cytokine release, degrade complement components and drive thrombo-inflammatory dysregulation. This review summarizes mechanistic insights into key microbial proteases such as EspP, Protease IV, LasB and SpeB, highlighting experimental models, diagnostic challenges and emerging protease-targeted therapeutic strategies with implications for improving sepsis outcomes.

## Introduction

Sepsis represents an intricate host-pathogen interaction between the host immune system and the invading pathogens, often resulting in multiorgan dysfunction and high mortality [1]. One of the central features in sepsis pathogenesis is the disruption of the vascular endothelial barrier, resulting in capillary leakage, hypotension and disseminated intravascular coagulation (DIC) [2]. During sepsis, the endothelial barrier, which controls vascular tone, permeability and coagulation is severely impaired.

Although, host-derived mediators of endothelial injury have been extensively studied, microbial proteases remain underexplored despite their direct role on endothelial integrity [3]. Bacterial proteases have the capacity to degrade structural proteins, disintegrate tight and adherent junctions and undermine the anticoagulant and anti-inflammatory activity of the endothelium [4]. These conditions, along with systemic inflammation, can accelerate endothelial dysfunction and predispose patients to the critical thrombo-inflammatory state, a typical trait of sepsis [5].

Globally, over 11 million deaths are caused by sepsis annually, reflecting the need to identify new pathogenic mechanisms and drug targets [6]. Despite many studies aimed at determining the role of cytokines and immune cells in endothelial injury, the facilitation of this process by bacterial proteases has received inadequate attention.

This review primarily focuses on microbial proteases as key drivers of endothelial barrier disruption and sepsis-associated coagulopathy, an aspect that remains underrepresented in the current sepsis literature. Specifically, it examines representative bacterial proteases, such as Protease IV and LasB from *Pseudomonas aeruginosa*, EspP from *Escherichia coli* and SpeB from *Streptococcus pyogenes*, highlighting their mechanisms of endothelial damage contributing to thrombo-inflammatory dysregulation in sepsis and implications for the therapeutic and diagnostic opportunities.

## The endothelium in sepsis

### Structure and function

The endothelium is a monolayer of specialized cells that line blood vessels and plays an active role in controlling vascular tone, permeability, inflammation and coagulation [7]. Endothelial cells are physically tethered together by junctional proteins such as vascular endothelial (VE)- cadherin, claudins, occludins and zonula occludens (ZO) proteins, creating tight and adherent junctions [4]. Furthermore, the endothelial surface is protected by a carbohydrate-layered structure called the glycocalyx, which consists of proteoglycans and glycoproteins and functions as a mechano-transducer, controlling leukocyte adhesion and vascular permeability [8]. In addition to increasing the vascular permeability, endothelial glycocalyx disruption exposes procoagulant surfaces and impairs anticoagulant signaling, which in turn contributes to coagulopathy.

### Pathophysiology in sepsis

Sepsis triggers a complex pathophysiological cascade characterized by systemic inflammation, endothelial dysfunction, immunological dysregulation and coagulopathy. The vascular endothelium serves as the gatekeeper, acting as the central hub of these processes and plays the role of both a mediator and a target of septic injury [9].

During infection, microbial molecules such as the lipopolysaccharide (LPS) of Gram-negative bacteria and lipoteichoic acid of Gram-positive bacteria are recognized by pattern recognition receptors (PRRs), i.e. Toll-like receptors (TLRs), on the endothelial and immune cells [10]. Ince et al. [11] reported that, this recognition triggers a robust inflammatory response, which is followed by the release of cytokines such as tumor necrosis factor-alpha (TNF-α), interleukin-1 beta (IL-1β) and interleukin-6 (IL-6).

These cytokines then activate endothelial cells and enhance the expression of adhesion molecules such as Intercellular Adhesion Molecule-1 (ICAM)-1 and Vascular Cell Adhesion Molecule-1 (VCAM)-1, which subsequently enhances leukocyte recruitment and transmigration.

Simultaneously, cytoskeletal reorganization occurs and causes damage to the protective glycocalyx layer and resulting in the disturbance of tight and adherent junctions, which destabilizes the endothelial barrier [4,8]. These changes increase the vascular permeability, which induces tissue edema, hypoperfusion and plasma leakage, all of which aggravate organ dysfunction.

In addition, there is an extreme imbalance in the coagulation system due to sepsis. Endothelial injury translates to increased tissue factor activation, leading to increased generation of thrombin and reduction in the concentration of anticoagulant molecules, such as thrombomodulin and the endothelial protein C receptor (EPCR) [12]. The ensuing procoagulant state may result in DIC, a symptom of far-reaching microvascular thrombosis, paradoxically resulting in the depletion of platelets and clotting factors and severe bleeding.

Notably, bacterial virulence factors such as proteases can exacerbate these processes. They, in combination with the host’s inflammatory response, according to Maeda [13], exacerbate endothelial damage and coagulopathy by directly breaking down endothelial junctional proteins and anticoagulant molecules. The microbial enzymes such as proteases are therefore centrally involved in the pathophysiology of sepsis, causing a destructive synergy between the microbial invasion, immunological activation, endothelial breakdown and coagulation dysfunction.

## Overview of bacterial proteases

Bacterial proteases are a diverse group of enzymes that are crucial for the survival, colonization and virulence of pathogenic bacteria. These secreted or membrane-bound enzymes break the peptide bonds in host and bacterial proteins. In addition to their fundamental role in the acquisition of nutrients, bacterial proteases are also involved in immunological modulation, disrupting barriers and dysregulation of coagulation and inflammatory processes, all of which are major hallmarks of sepsis pathogenesis [13].

Importantly, the bacterial proteases listed in (Table 1) are chosen based on experimental evidence of direct effects on endothelial structure, glycocalyx integrity and host coagulation pathways, linking bacterial protease activity to vascular leakage, immunopathology and sepsis-associated coagulopathy, rather than the general bacterial virulence alone. Due to variance in catalytic mechanisms and substrate specificity, these enzymes cause a variety of pathogenic consequences during bloodstream infections and septic shock. Other protease classes, such as aminopeptidases and aspartic proteases, may also play a role in host – pathogen interactions during sepsis, although this review mainly focuses on representative serine proteases, metalloproteases, cysteine proteases and carboxypeptidases with established roles in endothelial dysfunction and coagulopathy.

**Table 1.** Classification of bacterial proteases involved in endothelial dysfunction and sepsis-associated coagulopathy.

Class	Protease	Source organism	Known substrates	Reported virulence role	References
Serine protease (SPATE)	EspP	*Escherichia coli* (STEC)	Coagulation factors, complement proteins	Disrupts coagulation, complement evasion	[14]
Serine protease	Protease IV	*Pseudomonas aeruginosa*	Fibrinogen, complement C3, plasminogen	Immune evasion, impaired clot formation	[15]
Metalloprotease	LasB(Elastase)	*Pseudomonas aeruginosa*	Elastin, fibrinogen, laminin	ECM degradation, vascular leakage	[16]
Cysteine proteases	SpeB	*Streptococcus pyogenes*	Fibrinogen, fibronectin, complement proteins	Prevents clot formation, Endothelium damage, immune evasion	[17]
Carboxypeptidases	LdcB	*Streptococcus pneumoniae*	Bacterial cell wall tetrapeptides	Immune evasion	[18]

### Classification of bacterial proteases

**Figure 1.**
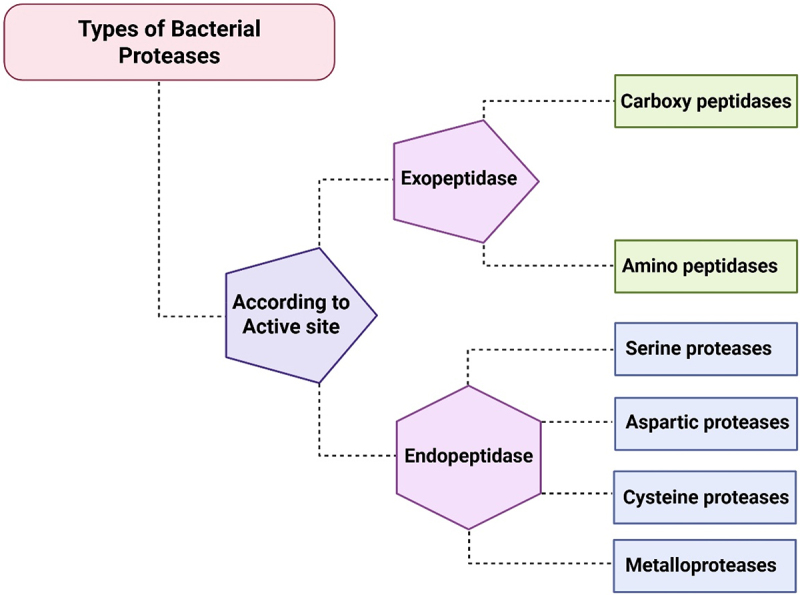
Classification of bacterial proteases (created using BioRender).

Although bacterial proteases can be classified on the basis of many criteria, they are broadly categorized into exopeptidases (which cleave peptide bonds near the ends i.e. N- or C- terminal residues of the protein chain) and endopeptidases (which cleave peptide bonds within the protein chain) on the basis of their site of action. Furthermore, they are classified on the basis of the type of active site residue that catalyzes the reaction (Figure 1) [17], with the major types involved in sepsis pathogenesis being:

- **Serine proteases**: Typically found in Gram-negative bacteria, these enzymes have a serine residue within their catalytic triad. Protease IV of *Pseudomonas aeruginosa* (*P.aeruginosa*) and EspP of *Escherichia coli* (*E.coli*) are two examples [14,19].
- **Metalloproteases**: Their mechanism relies on divalent metal ions (usually zinc) for catalysis. An example that acts against structural and immune-related proteins is LasB (also known as pseudolysin) from *Pseudomonas aeruginosa* [17,20].
- **Cysteine proteases**: This type of protease utilizes a cysteine residue to catalyze the reactions. An example that causes endothelial damage and immune evasion is SpeB of *Streptococcus pyogenes* (*S.pyogenes*) [17].

**Aspartic proteases**, along with other categories exist; however, only a few data regarding their function in vascular and immune disruption in sepsis are available.

Collectively, these proteases exhibit distinct but overlapping pathogenic specificities, with some preferentially targeting coagulation factors, others primarily disrupting endothelial barrier integrity and several influencing both processes, thereby reinforcing their central role in sepsis-associated coagulopathy.

The major classes of bacterial proteases implicated in sepsis pathogenesis include serine proteases, aspartic proteases, cysteine proteases, metalloproteases, carboxypeptidases and aminopeptidases. Representative examples from clinically relevant pathogens are shown, highlighting differences in catalytic mechanisms and substrate specificity. Many of these proteases contribute to bacterial virulence by targeting host structural proteins, immune components and coagulation factors, thereby promoting endothelial dysfunction and coagulopathy during sepsis.

### General virulence roles

Proteases are among the most versatile bacterial virulence factors, affecting various stages of infection through direct and indirect mechanisms, such as:

- **Tissue invasion**: Bacterial proteases hydrolyze extracellular matrix (ECM) proteins such as collagen, fibronectin and laminin to facilitate penetration and spread into deeper tissues [13].
- **Immune evasion**: By inactivating complement proteins (C3, C5), antibodies (IgA, IgG) and antimicrobial peptides, proteases may dampen the host immunological response. For example, the aureolysin of *Staphylococcus aureus* cleaves complement C3, reducing phagocytosis and opsonization [21].
- **Endothelial disruption**: Proteases may increase vascular permeability and leakage by directly cleaving endothelial junctional proteins, such as occludin and VE-cadherin or by degrading protective glycocalyx components [8].
- **Coagulation interference**: LasB and SpeB break the thrombomodulin and inhibit protein C activation, while other proteases like EspP, cleaves coagulation factor V, resulting in interference with the coagulation cascade and increases bleeding tendency [14,17].
- **Inflammatory amplification**: Proteases may trigger either toll-like receptors (TLRs) or protease-activated receptors (PARs), that may induce cytokine production and neutrophil activation and lead to systemic inflammation [10].

Together, these features allow bacteria to survive, spread and cause host tissue damage, the combination that is particularly fatal in sepsis.

## Mechanism of endothelium damage by proteases

The integrity of the vascular endothelium is crucial for controlling hemostasis, vascular permeability and immune homeostasis. However, this endothelial function is disrupted during sepsis by host-derived and microbial mediators that act in concert. Among microbial factors, bacterial proteases play a vital role in the pathogenesis of vascular leakage, coagulopathy and organ dysfunction through both direct and indirect mechanisms. The key among these factors is the degradation of endothelial junctional proteins, the cleavage of anticoagulant molecules and the enhancement of the inflammatory signaling pathway.

### Direct cleavage of junction proteins

The integrity of the endothelial barrier is maintained by two major types of endothelial junctions- adherent junctions (formed by VE-cadherin) and tight junctions (formed by proteins like occludin, claudins and ZO proteins), which together constitute a selective barrier between the bloodstream and surrounding tissues. Degradation of these complexes leads to vascular leakage, edema and organ failure, which are all the central hallmarks of sepsis.

Some experimental studies have demonstrated that microbial proteases directly damage intercellular junctions, which is essential for the endothelial barrier integrity. Schmidt et al. [8] demonstrated this mechanism via intravital confocal microscopy in endotoxemic mice. Within hours of administering LPS, researchers observed rapid, patchy shedding of the endothelial glycocalyx, along with increased albumin extravasation and dysregulated neutrophil adhesion. The structural loss of junctional organization was not solely architectural, it also resulted in a functional collapse of barrier integrity, leading to early vascular leakage and allowing the pathogens to invade immune cells and cause uncontrolled inflammation.

Several bacterial enzymes have been shown to cleave junctional proteins directly via proteolysis. Maeda [13] reported that LasB, a zinc-metalloprotease from *Pseudomonas aeruginosa*, cleaves both occludin and VE-cadherin, resulting in prominent gap formation and significant loss of endothelial integrity. Similarly, *Escherichia coli* secretes serine proteases such as EspP, which destroy cytoskeletal elements like actin filaments [14], thereby destabilizing adherens junctions and indirectly affecting barrier integrity. These events collectively result in sepsis-induced edema, hypotension and tissue hypoxia.

They reported a significant reduction in trans-endothelial electrical resistance within 60 minutes, indicating an abrupt loss of tight junction function. Immunofluorescence imaging revealed the degradation of VE-cadherin belts and disassembly of ZO-1 in cell borders, providing clear molecular evidence that bacterial proteases degrade the adhesion complexes required for endothelial cohesion.

Translating these mechanistic insights to clinical settings, Becker et al. [4] expanded these findings to human samples, revealing that individuals with severe sepsis had significantly higher levels of syndecan-1, a biomarker of glycocalyx degradation. These findings correlated with the glycocalyx breakdown with an increase in Intensive Care Unit (ICU) mortality, highlighting the clinical significance of protease-mediated junctional disruption.

### Degradation of anticoagulant surface molecules

The endothelium ensures blood fluidity and clot formation by synthesizing key anticoagulant proteins, such as thrombomodulin, the endothelial protein C receptor (EPCR) and tissue factor pathway inhibitor (TFPI). However, bacterial proteases disrupt this balance by cleaving these molecules and converting the endothelial surface from an anticoagulant state into a procoagulant state.

Ammollo et al. [5], reported that exposing endothelial cells to extracellular histones resulted in quick internalization and cleavage of thrombomodulin, resulting in complete loss of protein C activation capability. While histones themselves are host-derived, the exposure of endothelial cells to protease-induced damage amplifies histone release and accelerates in the loss of anticoagulant molecules. This function creates an amplifying feedback loop in which protease-mediated damage promotes histone shedding, which further erodes the endothelial anticoagulant phenotype.

In addition, numerous bacterial proteases directly interact with anticoagulant and coagulation pathways, as summarized in (Table 2). These enzymes act on fibrinogen, thrombin, coagulation factors and junction-associated proteins, affecting clot formation, disrupting endothelial surfaces and encouraging microvascular thrombosis or bleeding. Rather than functioning separately, these protease-driven mechanisms work together to degrade the endothelium’s anticoagulant surface, hence maintaining the thrombo-inflammatory cycle that defines sepsis-associated coagulopathy.

**Table 2.** Protease-specific effects in sepsis.

Protease	Endothelial target	Mechanism of action	Functional outcome	References
SpeB	Fibrinogen, fibronectin, laminin, pro–IL-1β	Proteolytic cleavage & cytokine activation	Barrier disruption, inflammation, impaired hemostasis	[17]
LasB	Laminin, thrombin, fibrinogen	ECM and coagulation factor degradation	Vascular leakage, bleeding	[22]
Protease IV	VE-cadherin, fibrinogen	Junction protein degradation	Barrier leakage, coagulopathy	[19])
EspP	Coagulation factor V, VIII, fibrinogen	Proteolytic cleavage	Impaired clot formation	[14,19]

### Inflammatory amplification

**Figure 2.**
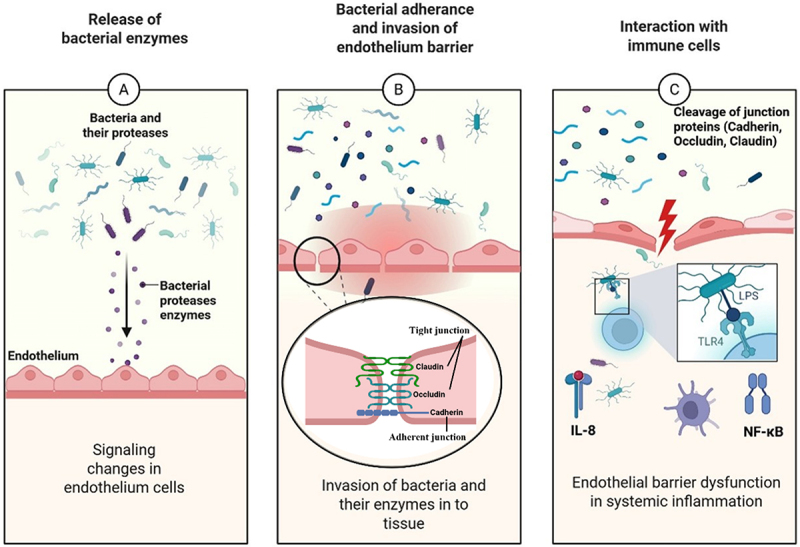
Mechanisms of endothelial barrier disruption by bacterial proteases (created using BioRender).

Bacterial proteases are capable of eliciting vigorous pro-inflammatory reactions as well as direct structural damage to host cells and tissues (Figure 2). Through their ability to activate host pattern recognition receptors including toll-like receptors (TLRs) and protease-activated receptors (PARs), they increase the production of cytokines, chemokines and damage-associated molecular patterns (DAMPs), which in turn, recruit and activate the neutrophils and the monocytes [5].

As stated by Maeda [13], LasB has been found to induce PAR-2 signaling in endothelial cells which results in calcium influx, NF-κB activation and IL-8 production, all of which are critical processes that sustain vascular inflammation. Furthermore, this inflammatory cascade is amplified as protease-induced destruction of the glycocalyx exposes endothelium surfaces, leading to increased leukocyte adhesion and subsequent inflammatory damage [4].

Ultimately, this protease-driven inflammation amplifies the host immune responses, which worsens the tissue damage, causing systemic endothelial dysfunction and leading to the widespread multi-organ failure observed in sepsis.

Collectively, the mechanism described in this section reveal how bacterial proteases contribute to endothelium dysfunction via several, interconnected routes. Direct cleavage of endothelial junctional proteins compromises barrier integrity, whereas degradation of anticoagulant surface molecules alters the endothelium toward a procoagulant state. In parallel, protease-mediated inflammatory amplification disrupts vascular homeostasis. Subsequently, this protease-driven inflammation increases host immunological responses, worsens tissue damage, causes systemic endothelial dysfunction and leads to the widespread multi-organ failure that occurs in sepsis.

Illustration depicting the principal mechanisms by which bacterial proteases compromise endothelial barrier integrity. Protease-mediated cleavage of intercellular junction proteins (VE-cadherin, occludin, claudins), degradation of the endothelial glycocalyx, inactivation of anticoagulant surface molecules and amplification of inflammatory signaling collectively result in increased vascular permeability, leukocyte adhesion and endothelial dysfunction. These events contribute to vascular leakage and thrombo-inflammatory dysregulation in sepsis.

## Protease-specific case studies

**Figure 3.**
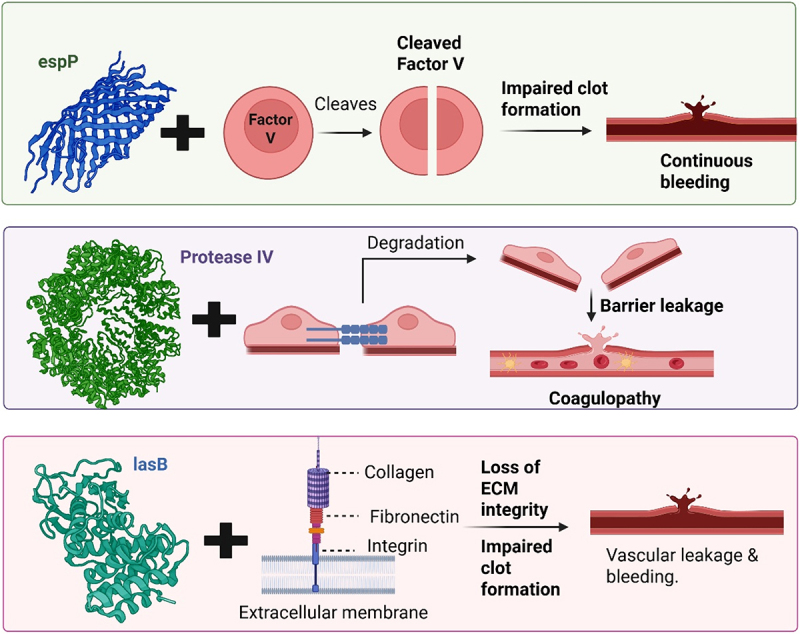
Protease-specific effects in sepsis (created using BioRender).

Understanding the function of each bacterial protease in endothelial dysfunction provides a mechanistic explanation for pathogen-specific variation in the severity of sepsis. This section profiles three of the most recognized virulence-associated proteases (Figure 3): EspP of *Escherichia coli* and Protease IV and LasB of *Pseudomonas aeruginosa*. While each enzyme possesses novel structural and functional features, they collectively contribute to the hallmarks of sepsis pathogenesis and result in the same pathogenic outcome i.e. loss of vascular integrity and facilitation of coagulopathy.

Representation of the distinct yet overlapping pathogenic actions of representative bacterial proteases, including EspP, which primarily targets coagulation factors and complement components; Protease IV affects junctional proteins and coagulation factors; LasB degrades extracellular matrix proteins and clotting factors. Together, these protease-specific activities drive endothelial injury, impaired hemostasis, immune evasion and progression of sepsis-associated coagulopathy.

### EspP– Escherichia coli

EspP (extracellular serine protease, plasmid-encoded) is a serine protease produced by enterohemorrhagic *Escherichia coli* (EHEC) via the type V secretion system [14]. It belongs to the SPATE (serine protease autotransporters of Enterobacteriaceae) family and plays a vital role in bacterial virulence. EspP has been shown to degrade coagulation factor V, an important cofactor in the prothrombinase complex that disrupts coagulation and enhances bleeding tendencies in infected hosts [23]. EspP also cleaves coagulation factor VIII, which might further contribute to coagulopathy.

Kuo et al. [23] demonstrated EspP-mediated coagulopathy; human plasma incubated with EspP showed a significant increase in PT, aPTT and TT. Using purified EspP, dose-dependent cleavage of Factor V, Factor VII, Factor VIII, Factor XII, prothrombin and fibrinogen was observed, which ended in a near-complete loss of functional fibrin polymerization. When whole blood thromboelastography (TEG) was carried out, EspP reduced the maximum amplitude (MA) of the clot while increasing the rate of clot lysis (LY30). These findings show that EspP alters hemostasis *in vitro* by decreasing clot strength and increasing fibrinolysis, giving additional evidence of the functional role of this protease in coagulopathy.

EspP is also involved in disrupting the endothelial barrier. *In vitro* studies of human brain microvascular endothelial cells (HBMECs) have demonstrated that exposure to EspP results in the disruption of the tight junctions and increased monolayer permeability [23,24]. In addition, EspP-induced cleavage of extracellular matrix components, such as laminin and fibronectin can disrupt endothelial integrity and facilitate systemic dissemination of bacteria.

Moreover, EspP facilitates immune evasion by degrading mucins, complement proteins and immunoglobulins. In particular, EspP cleaves the key complement components C3/C3b and C5, impairing opsonization and inhibiting the production of the major chemotactic factors C3a and C5a [20]. This mechanism is critical for immune evasion because it hinders opsonization (marks the bacteria for death) and antibody-mediated clearance, allowing the pathogen to survive and exacerbate the systemic inflammatory response. The multifactorial activities of EspP suggest that it facilitates not only bacterial invasion but also the coagulation dysfunction associated with coagulopathy and endothelial breakdown, both of which are characteristics of sepsis.

### Protease IV– Pseudomonas aeruginosa

Protease IV (PIV) is a lysine-specific serine endopeptidase that is secreted by *Pseudomonas aeruginosa* via the type II secretion system. It has been implicated in many host-pathogen interaction mechanisms, particularly tissue destruction and immune system evasion. Protease IV inhibits coagulation and inflammatory responses by cleaving fibrinogen, complement proteins and plasminogen [15,19].

According to Kida et al. [19], PIV is strongly associated with the abolishment of endothelial barrier integrity, they demonstrated this in mouse models of sepsis and pneumonia, where the PIV activity resulted in hemorrhagic lesions and vascular leakage in the lung. Proteases have also been implicated in degrading antimicrobial peptides and surfactant proteins, thus further reducing host defense mechanisms [25]. Interestingly, PIV directly degrades surfactant proteins A and D in the lung, neutralizing key opsonins and host defenses. It may also affect the inflammatory response by degrading cytokines such as interleukin-22 (IL-22), thereby hindering the production of local antimicrobial peptides [26]. In addition, *in vitro* studies have demonstrated that Protease IV is involved in direct junctional protein degradation by reducing the expression ZO-1 and VE-cadherin on the endothelium [19].

Bradshaw et al. [27] reported that PIV depletes IL-22 in the *in vivo* models, a cytokine required for epithelial barrier integrity and host defense in the lungs. This depletion weakens the mucosal barrier, which exacerbates the systemic inflammatory response (SIRS). This increased infection is a major upstream trigger for extensive endothelial dysfunction and abnormal activation of the coagulation cascade, connecting PIV’s immunomodulatory activity to the development of sepsis and coagulopathy.

These multifaceted activities strongly link Protease IV as a key factor in *Pseudomonas aeruginosa*-mediated coagulopathy and endothelial dysfunction, highlighting its potential as a therapeutic target.

### LasB– Pseudomonas aeruginosa

Pseudolysin or LasB, is a zinc dependent metalloprotease secreted by *Pseudomonas aeruginosa*. It is a critical virulence factor for both acute and chronic infections and possesses broad substrate specificity. Multiple host proteins such as fibrinogen, thrombin, tissue factor pathway inhibitor (TFPI) and extracellular matrix components are cleaved by LasB [16].

In infected tissues, LasB mediated cleavage of thrombin and fibrinogen interferes with clot stabilization and increases the tendency for bleeding. LasB is also involved in immune evasion through the inactivation of cytokines, immunoglobulins and complement components (C3, C5) [28].

LasB is also linked to immune evasion by deactivating cytokines, immunoglobulins and complement components (C3, C5). LasB (elastase) and alkaline protease (AP) inactivates the key pro-inflammatory cytokines including Interferon (IFN) and TNF by limited proteolysis, although IL-1 is resistant to these proteases. This selective reduction in key immune mediators is likely to profoundly impact host immunological responses, leading to the immunosuppressive state as observed in chronic *Pseudomonas aeruginosa* infections [16].

Previous study reported that Saint-Criq et al. [29], LasB degrades epithelial immune mediators IL-6 and trappin-2 while inhibiting epithelial cell repair. This local damage is significant because the impaired respiratory barrier promotes bacterial translocation and overwhelming systemic inflammation, which is the decisive upstream trigger for widespread endothelial dysfunction and pathological coagulopathy (DIC) in sepsis. As a result, targeting LasB or increasing protective factors such as IL-6 or trappin-2 provides a promising therapeutic target in *Pseudomonas aeruginosa* infected people.

Purified LasB alone caused significant lung damage *in vivo* and induced substantial degradation of extracellular matrix components and key proteins involved in the coagulation cascade without directly causing cellular death. LasB has also been reported to cause DIC-like coagulopathy *in vitro*. Intratracheal instillation of LasB in animal models resulted in pulmonary hemorrhage and acute lung injury, further supporting its role in sepsis-associated vascular leakage and DIC-like coagulopathy [30].

Together, the case studies of EspP, Protease IV and LasB highlight targeted enzymatic degradation as the primary mechanism through which these bacterial proteases cause sepsis. All three are important virulence factors that converge on the same clinical outcomes while having distinctive structural features. However, they simultaneously damage the vascular endothelium (by cleaving junctional or matrix proteins) and disrupt systemic hemostasis (by cleaving coagulation factors and fibrinogen). This potent dual-action mechanism combines pro-hemorrhagic coagulopathy with direct vascular damage, which is crucial to the severity of sepsis and supports the focus on these enzymes as suitable therapeutic targets for anti-virulence therapies.

## Experimental models and assays to study bacterial proteases in sepsis

**Figure 4.**
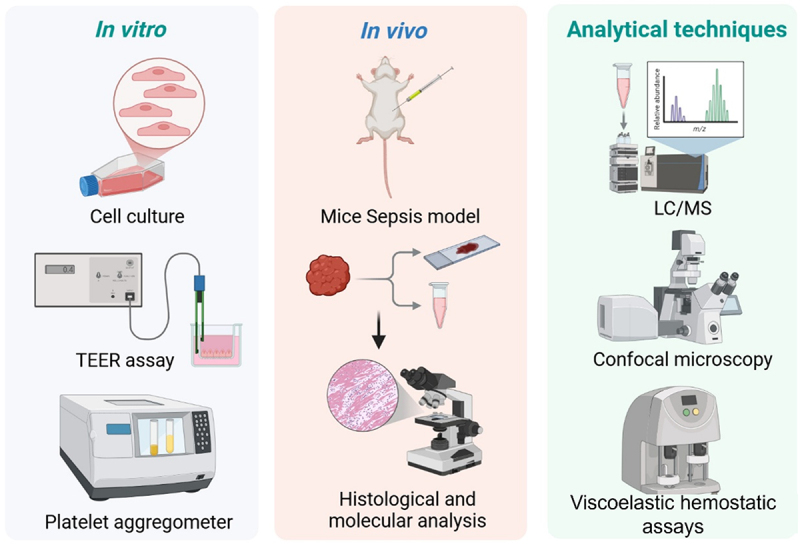
Experimental model’s pipeline (created using BioRender).

The use of the complementary *in vitro, in vivo* and analytical methods is necessary to understand the role of bacterial proteases in coagulopathy in sepsis (Figure 4). These models enable the analysis of host-pathogen interactions, molecular pathways and validation of the therapeutic approaches.

Illustrating the integrated experimental approaches used to investigate bacterial protease – mediated endothelial dysfunction and coagulopathy. The pipeline includes *in vitro* endothelial and coagulation assays, *in vivo* sepsis model and protease-injection models and complementary analytical techniques such as proteomics, imaging and functional coagulation measurements. Each model provides distinct but complementary insights into protease activity, mechanism of action and therapeutic targeting.

### In vitro models

The benefit of *in vitro* systems is that they offer controlled conditions where certain cell types or plasma can be directly exposed to purified bacterial proteases such as Protease IV (*P. aeruginosa*) and EspP (*E. coli*). They are necessary for the separation of protease-specific effects from those of other bacterial virulence factors [31].

#### Endothelial cell cultures

- To analyze the proteolytic breakdown of junctional proteins (VE-cadherin, claudin-5, occludin) and surface anticoagulant molecules (thrombomodulin, endothelial protein C receptor), primary human umbilical vein endothelial cells (HUVECs) and human microvascular endothelial cells (HMEC-1) are used.
- To measure the strength, permeability and integrity of cell barriers, trans-endothelial electrical resistance (TEER) or FITC – dextran permeability assays are employed since these methods provide complementary methods following exposure to proteases [32].
- Confocal microscopy may be employed to visualize cytoskeletal rearrangements and glycocalyx degradation (syndecan-1 shedding) and ELISA can be used to quantify these changes [33]. Western blotting of cell lysates confirms junctional protein cleavage at the molecular level.

#### Platelet function assays

- The treatment of washed platelets or platelet-rich plasma with proteases may accomplish the evaluation of aggregation kinetics by optical aggregometry. The use of washed platelets minimizes interference from plasma components, ensuring that the effect is a direct protease-platelet interaction.
- Activation markers (P-selectin and activated GPIIb/IIIa), following protease treatment, can be quantified by flow cytometry [34].

These assays clarify whether proteases affect clotting indirectly by cleaving fibrinogen or directly by inhibiting platelet function.

#### Plasma coagulation assays

- Performing activated partial thromboplastin time (aPTT) and prothrombin time (PT) assays reveals the extrinsic or intrinsic pathways of protease-mediated clotting factor degradation [23].
- One can ascertain whether EspP or Protease IV selectively cleaves thrombin, fibrinogen, factor V, factor VIII or X using factor-specific chromogenic assays.

##### Strengths and limitations of *in vitro* models

*In vitro* systems provide a high degree of experimental control, allowing for exact examination of protease-specific effects on endothelial integrity, platelet function and coagulation pathways. These models are especially useful for identifying direct protease-host interactions without confounding from immunological responses or bacterial replication. However, their primary drawback is the lack of systemic factors like immune cell recruitment, hemodynamic shear stress and organ-level interactions, which limit their capacity to accurately recapitulate the complex pathophysiology of sepsis.

### *In vivo* models

The dynamic interplay among coagulation dysregulation, immune activation and bacterial infection is elucidated using animal models. They are crucial in understanding the systemic effects of protease action in sepsis [35].

#### Murine sepsis models

- Cecal ligation and puncture (CLP) causes polymicrobial sepsis with coagulopathy symptoms similar to those of human DIC.
- Survival, organ histopathology and coagulation parameters can all be compared between protease-producing and protease-deficient bacterial strains [35].
- The role of isogenic bacterial strains with different levels of protease expression in coagulopathy can be confirmed directly by intravenous or intraperitoneal bacterial injection, allowing controlled monomicrobial infection studies.

#### Knockout models with specific gene deletions

Protein C or fibrinogen-deficient mice can be used to determine whether protease activity targets specific coagulation pathways.

Vascular injury caused by protease can be visualized in real time in transgenic mice, that secretes the fluorescent fibrinogen or endothelial junction proteins.

#### Protease injection models

Purified EspP or Protease IV can be injected directly into the bloodstream or tissue to monitor acute vascular leakage, fibrinolysis and depletion of clotting factors without affecting bacterial growth [19].

Acute vascular leakage is measured by the extravasated dye (e.g. Evans blue or FITC-dextran) recovered from homogenized tissue, thereby linking the macroscopic observations to a measurable value. Dose-response analysis and therapeutic neutralization tests (e.g. protease inhibitors, antibodies) are facilitated by this approach.

##### Strengths and limitations of *in vitro* models

*In vivo* sepsis models capture the complex interaction of bacterial pathogenicity, endothelial dysfunction, inflammation and coagulation disorders, providing greater physiological relevance than *in vitro* systems. Models such as CLP and pathogen-specific infections can be used to examine vascular leakage, disseminated intravascular coagulation, organ damage and survival outcomes. However, species-specific differences in immune and coagulation systems, variability in disease severity and ethical constraints limits direct extrapolation to human sepsis. Protease injection models enhance mechanistic specificity, but they do not fully capture the dynamics of bacterial infection and host-pathogen interaction.

### Analytical techniques

Sensitive analytical techniques are needed to characterize protease activity and monitor its downstream impact on the coagulation system.

#### Proteomics

LC-MS/MS degradomics confirms target specificity by determining cleavage sites of coagulation proteins [36]. This method is superior for determining the precise amino acid cleavage site (P1-P1’ bond), which helps define the protease’s substrate specificity and mechanism of action.

Quantitative proteomics can track the slow decline in the plasma levels of clotting factors following exposure to proteases for various periods of time.

#### Zymography and enzymatic assays

Protease molecular masses and activity profiles in bacterial supernatants or infected tissue homogenates can be resolved using gelatin or casein zymography, as described in the literature [37].

Inhibitor screening is enabled by high-throughput fluorogenic substrate assays that provide real-time measurement of serine protease activity [38,39].

#### Imaging techniques

- Glycocalyx loss and disruption of endothelial junctions can be observed by confocal microscopy [40].
- Architectural changes in fibrin within clots treated with bacterial protease are observable with scanning electron microscopy (SEM).

#### Functional coagulation measurements

Dynamic clot formation and lysis patterns are determined by viscoelastic hemostatic assays such as thromboelastography (TEG), which measures both procoagulant and fibrinolytic effects [41]. The essential metrics (R time, K time, α angle and Maximum amplitude) identify hypercoagulability, hypocoagulability and hyperfibrinolysis.

##### Strengths and limitations of analytical approaches

Each analytical technique has distinct advantages for studying protease-mediated coagulopathy, but it also has inherent shortcomings. Proteomics-based approaches enable highly sensitive and unbiased identification of protease substrates and cleavage sites, but they require specialized apparatus, sophisticated bioinformatics skills and are not easily adapted to routine clinical application. Zymography and enzymatic assays are inexpensive and useful for activity profiling and inhibitor screening; however, they are semi-quantitative and rely on substrate selection. Confocal microscopy and scanning electron microscopy are imaging methods that allow for direct detection of endothelial junction rupture, glycocalyx loss and fibrin architecture, however they are mostly descriptive and are of low-throughput. Functional coagulation measurements, such as thromboelastography, incorporate procoagulant and fibrinolytic effects in real time and provide therapeutically useful insights, but they do not identify particular protease-substrate interactions that cause the observed coagulation anomalies. As a result, a combinatorial analytical approach is required for a comprehensive characterization of bacterial protease-driven coagulation dysfunction in sepsis.

## Clinical relevance and therapeutic directions

Bacterial proteases play a dual and clinically significant role in the pathophysiology of sepsis: they cause direct damage to the vascular endothelium and indirectly exacerbate the host’s coagulopathic and inflammatory responses. Clinically, their presence has been linked with increased mortality rates, accelerated progression of disseminated intravascular coagulation (DIC) and greater severity of organ dysfunction [42]. Several clinical studies have shown that the detection of protease activity in patient specimens like plasma, bronchoalveolar lavage fluid or wound exudate can serve as an early biomarker for endothelial damage and predict patient outcomes more reliably than the conventional markers [39]. These results indicate that microbial proteases are an active cause of vascular damage and coagulopathy in sepsis rather than just byproducts of infection.

Several approaches for neutralizing bacterial protease activity are currently under investigation (Table 3). These approaches broadly focus on inhibiting protease catalytic activity, preventing substrate interaction, or enhancing endothelial resilience, indicating a shift toward antivirulence-based interventions rather than direct bacterial killing in an attempt to limit endothelial injury and sepsis-associated coagulopathy.

**Table 3.** Therapeutic strategies targeting bacterial proteases.

Strategy	Target	Mechanism of action	References
Protease inhibitors (e.g. serine protease inhibitors)	EspP, Protease IV	Block the active site	[15,22]
Metal chelators (e.g. EDTA analogs)	Metalloproteases like LasB	Inhibit zinc-dependent catalysis	[35]
Neutralizing antibodies	EspP, Protease IV	Prevent substrate interaction	[15]
Small-molecule inhibitors	Specific protease pockets	Competitive/non-competitive inhibition	[35]
Vaccine-based approaches	Protease antigens	Induce neutralizing antibody response	[37]

One significant advantage of this antivirulence strategy is that it is expected to minimize the evolutionary drive for antibiotic resistance. Endothelial protectants like sphingosine-1-phosphate modulators [43] and activated protein C analogues [44], are some adjuvant modalities that may preserve endothelial integrity. To avoid permanent endothelium damage and uncontrolled coagulation activation, early initiation of protease-targeting is crucial in the course of this disease [11,42]. These targeted therapeutic interventions and rapid point-of-care measurement of protease activity must be combined in future prospective clinical studies.

Despite these advancements, several critical research gaps currently hinder the clinical translation of protease-targeted therapies in sepsis:

- There is a lack of standardized, rapid bedside assays to quantify bacterial protease activity in septic patients.
- Limited human studies that directly correlate circulating protease levels with endothelial biomarkers, coagulation parameters and clinical outcomes.
- Insufficient understanding of dynamics of protease activity during different stages of sepsis.
- A lack of well-designed clinical trials evaluating the safety and efficacy of protease-directed therapies in sepsis.

## Conclusion and future perspectives

**Figure 5.**
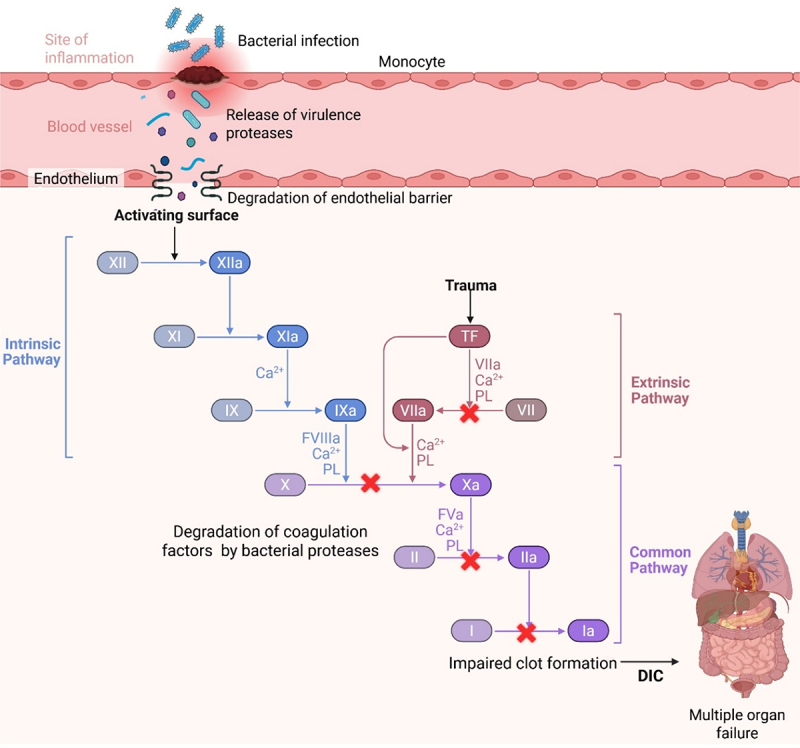
Protease-driven endothelial and coagulation dysregulation in sepsis (created using BioRender).

Microbial proteases remain an unrecognized yet pivotal players in the evolution of sepsis-associated endothelial dysfunction and coagulopathy. Their unique capacity to degrade structural junctional proteins, inactivate anticoagulant molecules, degrade coagulation factors and aggravate inflammatory cascades (Figure 5) firmly positions them as both mechanistic drivers of disease and potent biomarkers and potential therapeutic targets. Despite the substantial progress made in understanding their molecular pathways in the *in vitro* and animal models [23,35], the translation of these findings into meaningful clinical applications still remains incomplete. The existing gap between mechanistic insight and bedside implementation reflects the absence of standardized diagnostic methods and protease-directed therapeutics in routine clinical care.

Conceptual model summarizing the pathophysiological cascade initiated by bacterial infection and protease release. Bacterial proteases disrupt the endothelial barrier integrity, degrade anticoagulant molecules and coagulation factors, amplify inflammatory signaling, and promote complement dysregulation. These interconnected processes culminate in vascular leakage, disseminated intravascular coagulation, microvascular thrombosis and multiorgan dysfunction, highlighting bacterial proteases as key drivers of sepsis severity.

To bridge this gap, future research must emphasize the following directions:

- Development of standardized, protease detection assays for incorporation into rapid clinical care.
- Design of highly selective, sensitive protease inhibitors with minimal side effects.
- Prospective long-term research to correlate protease levels with the course of the disease and its outcomes is needed.
- Combination therapeutic strategies integrating protease inhibition with endothelial-protective, anti-inflammatory and antibacterial approaches.

Implementing these strategies, opens the door for a paradigm shift in sepsis management, from late-stage supportive care to early, mechanism guided interventions that target the key bacterial virulence factors. Interrupting protease driven vascular injury at its earliest stage, may minimize mortality, prevent permanent organ damage and drastically improve clinical outcomes in patients with sepsis.

## Data Availability

No data was used for the research described in the article.
